# Extracellular Vesicles and Autophagy in Osteoarthritis

**DOI:** 10.1155/2016/2428915

**Published:** 2016-12-18

**Authors:** Tianyang Gao, Weimin Guo, Mingxue Chen, Jingxiang Huang, Zhiguo Yuan, Yu Zhang, Mingjie Wang, Penghao Li, Jiang Peng, Aiyuan Wang, Yu Wang, Xiang Sui, Li Zhang, Wenjing Xu, Shibi Lu, Xifeng Zhang, Shuyun Liu, Quanyi Guo

**Affiliations:** ^1^Institute of Orthopaedics, Chinese PLA General Hospital, Beijing Key Lab of Regenerative Medicine in Orthopaedics, Key Laboratory of Musculoskeletal Trauma & War Injuries, 28 Fuxing Road, Haidian District, Beijing 100853, China; ^2^Shanxi Medical University, No. 56 XinJiannan Road, YingZe District, Taiyuan 030001, China; ^3^Minimal Invasive Spine Surgery Center, Spine Division of Orthopaedic Department, Chinese PLA General Hospital, 28 Fuxing Road, Haidian District, Beijing 100853, China

## Abstract

Osteoarthritis (OA) is a type of chronic joint disease that is characterized by the degeneration and loss of articular cartilage and hyperplasia of the synovium and subchondral bone. There is reasonable knowledge about articular cartilage physiology, biochemistry, and chondrocyte metabolism. However, the etiology and pathogenesis of OA remain unclear and need urgent clarification to guide the early diagnosis and treatment of OA. Extracellular vesicles (EVs) are small membrane-linking particles that are released from cells. In recent decades, several special biological properties have been found in EV, especially in terms of cartilage. Autophagy plays a critical role in the regulation of cellular homeostasis. Likewise, more and more research has gradually focused on the effect of autophagy on chondrocyte proliferation and function in OA. The synthesis and release of EV are closely associated with autophagy. At the same time, both EV and autophagy play a role in OA development. Based on the mechanism of EV and autophagy in OA development, EV may be beneficial in the early diagnosis of OA; on the other hand, the combination of EV and autophagy-related regulatory drugs may provide insight into possible OA therapeutic strategies.

## 1. Introduction

As the only cell type in adult articular cartilage, chondrocytes are terminally differentiated cells that have limited ability for cell proliferation [1]. Chondrocytes can respond to matrix structure changes of the surrounding cartilage, but the capacity to restore the normal cartilage matrix structure is limited. However, this capacity will gradually weaken with increasing age [2]. The metabolic homeostasis of chondrocytes is disrupted in osteoarthritis (OA) patients [3] and then continues to cause cartilage damage, intraarticular synovitis, subchondral bone changes, and joint pain. Homeostasis destruction can be reflected in biomechanics and biochemistry. The pathogenesis of OA may be due to articulated biomechanical changes, joint trauma, cytokines, or the immune response. However, the specific pathogenesis of OA has not yet been completely explained. As a current hot research topic, extracellular vesicles (EVs) and autophagy have been found to be associated with the occurrence and development of OA. Therefore, this paper will review the EV and autophagic mechanisms in OA, as well as potential clinical applications.

EVs are small membrane-linking particles that are released from cells, and they can be found in all tissues, including the synovium [4] and blood [5]. EV can be separated from the cell membrane by direct budding off or can be released after the fusion of endosomal multivesicular bodies with the plasma membrane. EV was first discovered in 1946, and EV was initially found in normal plasma-clotting platelet-derived particles [6]. In 1967, EV was described by Wolf as platelet waste production and as having no function [7]. However, it was found to play a role in the process of bone calcification [8] through some studies in 1969. In 1987, the ultrastructure of EV displayed that EV can be released through the fusion of multivesicular bodies with cell membranes [9]; in 1996, the term “exosome” was created to describe some types of EV [10]. Scientists have found that EV contains RNA, indicating that they are a carrier for communicating genetic information between cells, since 2006 [11]. EV can be broadly classified into three categories [12]. The first type, microvesicles/microparticles/ectosomes (MV), produced by budding from the plasma membrane or fission, has a diameter of 100–1000 nm. The second type, exosomes (EXOs), are produced through the fusion of multivesicular bodies with a diameter less than 100 nm. The third type, apoptotic bodies (ABs), comprises vesicles released during the process of apoptosis. However, it is impossible to just classify EV according to a single characteristic, such as size or structure. The community attempt to identify the EV specific subtype based on the novel isolation methods. Kowal et al. propose that the EVs can be distinguished as follows: (i) large EVs pelleting at low speed (ultracentrifugation pellet 2,000 ×g), (ii) medium-sized EVs pelleting at intermediate speed (10,000 ×g), and (iii) sEVs pelleting at high speed (10,000 ×g). Among the sEVs, four subcategories can be defined: (iiia) sEVs coenriched in CD63, CD9, and CD81 tetraspanins and endosome markers; (iiib) sEVs devoid of CD63 and CD81 but enriched in CD9; (iiic) sEVs devoid of CD63/CD9/CD81; and (iiid) sEVs enriched in extracellular matrix (ECM) or serum-derived factors [13].

EVs are not only biologically active signal molecule carriers (e.g., proteins, enzymes, mRNA, miRNA (microRNA), DNA, and lipids) but also contain some ligands on their cell membrane. On the one hand, EV can function indirectly through the signal path on the cell membrane. On the other hand, it can also be fused with the cell membrane and then transfer signaling molecules into the cytoplasm, thereby directly activating or inhibiting specific intracellular activities. In addition, EVs can enter the cell by endocytosis and release their contents into the targeted organelle. As a very effective carrier in various biological processes, EVs participate in the information exchange between cells, sometimes with an extensive reach [14]. This review mainly discusses EV in terms of MV and EXO. The MV is the start position of calcification in all bone tissue, including growth plate cartilage. It is generated from polarized budding from the lateral edges of plate chondrocytes [15]. EXO can regulate or mediate the biological activities between cells, cell kinetics, and microenvironment homeostasis in cells as intercellular communication carriers. EXO can exchange information from different cell types using not only endogenous substances but also exogenous signaling molecules.

In addition to EV, autophagy is involved in the information exchange between cells and disease development, and it plays an important role in maintaining normal cellular homeostasis and microenvironment [16]. Autophagy is a continuous and steady degradation of cellular homeostasis that involves the formation of the autophagosome. The autophagosome will clear unwanted proteins and dysfunctional organelles that are harmful to cell function through phagocytosis, and then the autophagosome containing some waste products fuses with lysosomes to clear these products and cellular components in the circulation. When the cellular autophagy mechanism is deficient, it can cause harmful substance accumulation, abnormal gene expression, and eventually lead to cell death. In some studies, researchers have found that inhibiting the expression of autophagy genes can cause cell death [17], indirectly giving us the evidence that autophagy can protect cells from the danger of death and maintain normal cell function and metabolism. In a human endothelial cell (EC) apoptosis-inducing experiment, the result indicates that autophagy participates in cell apoptosis. Apart from AB, EC secretes another type of EV, different from AB. Unlike AB, the content of this EV did not contain numerous nuclear components. By contrast, it is rich in mitochondrial and autophagic composition, and its secretion is related to the ROCK1 (Rho-associated protein kinase 1) signaling pathway [18].

## 2. EV in OA

As early as 1969, a special type of EV generated in a growth plate in the developmental phase was found to be in the start calcification position of entochondrostosis. In this process, EV from chondroblasts and osteoblasts collected inorganic phosphate and calcium from the ECM, followed by mineralization in the lumen to form hydroxyapatite crystals. Next, hydroxyapatite crystal deposition in the ECM was mineralized further. Ultimately, the process of chondrogenesis is completed. Given the various growth factors and proteins found in EVs, including BMP (Bone Morphogenetic Protein) and VEGF (vascular endothelial growth factor) [19], EVs were speculated to also participate in blood vessel formation and the differentiation of chondrocytes and osteoblasts in growth plates. Articular cartilage vesicles (ACVs) are membrane-linking EVs that are secreted from normal cartilage, with a diameter between 50 nm and 250 nm, including MV and EXO. ACVs were first found in OA patients' articular cartilage [20] and were then observed in normal articular cartilage [21]. Cytoskeletal components, including actin and actin proteins, are also present in ACV. It was confirmed that ACVs are generated by budding from chondrocytes. Osteoblast lineage cells also secrete EV, but the components of these EVs are different from those of ACVs [22], indicating that different cell types will produce different EV.

In normal human articular cartilage, there are some differences between the proteins in ACV and membrane-limited particles. However, there are some similar protein characteristics in ACV and EV that originate from growth plate cartilage. Therefore, it can be considered that ACVs are the mineralization center of epiphyseal cartilage. The functions of ACV in normal articular cartilage include repairing the pericellular ECM around chondrocytes and neutralizing potentially toxic substances (e.g., ATP, calcium, and phosphorylation) that may be harmful to adjacent chondrocytes.

ACVs secreted from OA chondrocytes participate in atypical protein secretion, the information exchange between cells, and pathological calcification. ACVs do not only form pathological calcium crystals in the articular cartilage [23] but can also affect the normal chondrocyte phenotype by transducing RNA and proteins [24]. Proteins in ACV include ECM components, phospholipid-binding proteins, enzymes, and cytoskeletal components (including actin). Among them, the immune proteins and serum complement component are unique in the ACV from OA patients. ACVs in OA patients show a decreasing content of proteoglycans in the matrix but an increasing content of TGF-*β*-mediated (transforming growth factor-*β*-mediated) *β*ig-H3, DEL-1 (developmental endothelial locus-1), vitronectin, and the serine protease HtrA1 [25]. This phenomenon indicates that the changes in OA patient articular cartilage can be observed according to the changes in the AVC components. In the early stage of OA, ACV released by matrix-degrading enzymes can directly react with chondrocytes, leading to chondrocyte hypertrophy. On the one hand, the pathological process of OA can affect the ACV release; on the other hand, ACV released by OA chondrocytes may in turn affect the normal chondrocytes and further promote OA development. In the OA development process, these two factors are mutually influential and interact with each other.

EV in the synovial fluid may be directly derived from the secretion of synoviocytes or chondrocytes, indirectly transformed from plasma [26]. Some EV-related mechanisms have been found to play a role in rheumatoid arthritis (RA)/OA development, such as the release of growth factors and chemokines [27–29]. Synovial-derived EVs are mainly detected in autoimmune diseases, such as RA and juvenile idiopathic arthritis [30]. Citrullinated proteins can also be detected in RA, OA, and other reactive arthritic diseases [31]. It was found that EV-related factors can cause arthritis [32]. Many citrullinated proteins can aggregate through EV or EV-related factors, and citrulline was considered to be an important autoantigen in RA. Therefore, we can conclude that, apart from OA, EV plays a critical role in autoimmune diseases, such as RA.

## 3. Autophagy Mechanism in OA

Autophagy plays a role in cellular degradation, and it is crucial for maintaining cell survival. To date, studies have shown that autophagy functions by regulating the dysfunctional organelles and proteins to protect the organism against normal or pathological cell senescence [33]. As age increases, basic autophagy activation is weakened and more and more macromolecular protein aggregates, eventually leading to the degenerative changes and loss of function in cells, or even apoptosis [34]. In articular cartilage, the role of autophagy in the maintenance of cellular homeostasis and function is particularly important, due to the low rate of chondrocyte proliferation. Autophagy is considered a key factor in the pathogenesis of OA. Recent studies have shown that, in OA patients, autophagy activation function is missing, thus leading to chondrocyte death and tissue destruction [35].

Homeostasis in the intracellular environment depends on interactions between cells, normal functions in organelles, necessary biological macromolecules, and normal biosynthesis functions. A common feature of degenerative diseases (including OA) is the accumulation of destructive macromolecules, which leads to the loss of the ECM, cell dysfunction, and death [36]. In OA, the term “Chondroptosis” [37] describes the death of chondrocytes in articular cartilage, and this process involves apoptosis and autophagy.

The functional deficiency of autophagy can lead to mitochondrion dysfunction and abnormal accumulation, further increasing the risk of OA. For example, because of a lack of effective mitochondrial coupling in OA, the reparation ability of articular cartilage is deficient. Moreover, studies have observed a large amount of reactive oxidants in pathological chondrocytes [38]. In addition, the increase in oxidative stress, reduction of chondrocyte proliferation, inflammation, and death of chondrocytes are all related to mitochondrial dysfunction. Therefore, there is no doubt that mitochondrial dysfunction plays an important role in OA pathogenesis. Autophagy can be activated to combat the dysfunction of the mitochondria in human chondrocytes. It is one of the indispensable regulatory mechanisms for intracellular homeostasis. Autophagy does not only regulate nutrient provision but can also play an important role in the removal of dysfunctional organelles and macromolecules, an activity that can be confirmed in OA [39]. Animal studies have indicated that the activation of autophagy can prevent cartilage from mechanical damage in OA [40].

## 4. Relationship between EV and Autophagy in OA

Some studies have found that ACV could be generated by pressure or apoptosis; meanwhile, OA was found to be related to apoptosis [41]. Therefore, ACVs generated by pressure or apoptosis were speculated to play a role in OA development. On the one hand, in endothelial cells, when autophagy and apoptosis are activated simultaneously, EV will be released by cellular stress; on the other hand, some studies have attributed the mineralization characteristic of MV to AB because they believe AB can mineralize and that this is the starting position of pathologic mineralization [42]. In short, the roles of EV, autophagy, and apoptosis in OA are complicated and unclear; however, it appears that there will be a mutual influence among them.

Other studies have found that ACVs are generated continuously rather than previously generated by cell destruction or cell death. Membrane blebbing is the early phenomenon of apoptosis [43], but the increased apoptotic cells will not increase the released number of ACVs. Chondrocytes after apoptosis induction cannot increase the overall vesicle protein content to achieve excessive calcification. Jaovisidha et al. also showed that ACVs isolated from apoptotic cells do not increase the degree of calcification [44]. Combined with the current evidence in the literature, we can confirm that ACVs are different from ABs. First, ABs are much larger than ACVs; second, typical apoptotic activators will not increase the number of ACVs; third, the content of AB and ACV differs; finally, the literature suggests that there exists a big difference in the features and function of ACV and AB [45]. Currently, little is known about which organelles participate in the formation of ACV.

Furthermore, the formation and release of ACV are also affected by the environment in which they exist. Whether from normal chondrocytes or apoptotic chondrocytes, the ability of isolated vesicles in mineralization was similar. MVs are separated from the normal cartilage of young people, although it comes from the normal intact matrix; however, it will only be mineralized by the way of separation. This phenomenon indicated that, in terms of the calcification ability of MV and AB, matrix integrity is one of the most important factors [46]. Additionally, the pathological characteristics of all joint diseases, including OA and RA, are similar; their common feature is the directly lost balance between synthesis and catabolism, undermining the integrity of the ECM. The lack of autophagy could cause disorders of intracellular homeostasis and ECM destruction. Regardless of the type of homeostasis imbalance, it will lead to soluble molecules (e.g., cytokines, growth factors, and enzymes) and changes in the synovial fluid, similar to the degree and content of EV changes. There is some evidence suggesting that pathological processes, such as cell death or damage, will promote EV formation. For instance, nutrient deficiency of human endothelial cells (EC) can evoke the abnormal secretion of vesicles, distinct from AB and bearing the surface markers of multivesicular bodies (MVBs) [47]. However, growing evidence has indicated that autophagy is related to ACV generation [48], and lack of autophagy can contribute to the development of OA [35, 36]. When the cellular microenvironment has nutritional deficiencies or is under hypoxic conditions, the signal of autophagy is significantly increased in chondrocytes. In normal chondrocytes, ACV contains the autophagy marker LC3-II, and the release of ACV is related to caspase-3 and the Rho/ROCK signal pathway [49], providing more evidence that ACV generation is associated with the autophagy mechanism and is simultaneously produced with autophagy.

Cell number and cell proliferation within tissue are closely related to cell apoptosis, and the activation or inhibition of survival signaling channels (including the Ras/Raf/MEK/ERK (MAPK) and PTEN/PI3K/AKT/mTOR) can significantly alter the cell proliferation rate [50], which is very important to damaged tissue healing in OA. For example, the mTOR-like receptor is an important part of inhibiting autophagy, which acts upstream of the Atg gene coding strands to regulate upstream of the coding strand of multiple signal channels such as PI3 kinase/AKT and AMT activation protein kinase [51–53]. Rapamycin can cause autophagy in various cell types and leads to the increasing release of EV, which depends on caspase-3 and Rho/ROCK. Based on pharmacology and gene research, it was found that autophagy inhibitor drugs can counteract the effect of rapamycin stimulating chondrocytes to produce ACV [44]. Combined with the phenomenon of the lack of an ACV response in OA patient chondrocytes to rapamycin, we can presume that it may have difficulty in ACV synthesis and release, and that this imbalance is related to autophagy deficiency.

## 5. Prospective Application of EV in OA

Recognizing that autophagy is a key factor in producing ACV is a necessary and important step in exploring the role of ACV in the regulation of cartilage health and repair. Moreover, aiming at slowing down or even preventing OA development, further understanding the intrinsic molecular mechanisms in OA may help us achieve precise treatment using suitable drugs.

## 6. EV as a Biomarker for Future OA Diagnose

EVs have a number of features, including a special composition, regulation and release mechanisms, and component protection features, making EVs and their content expected novel biomarkers of OA pathology and pathophysiology.

**Figure 1:**
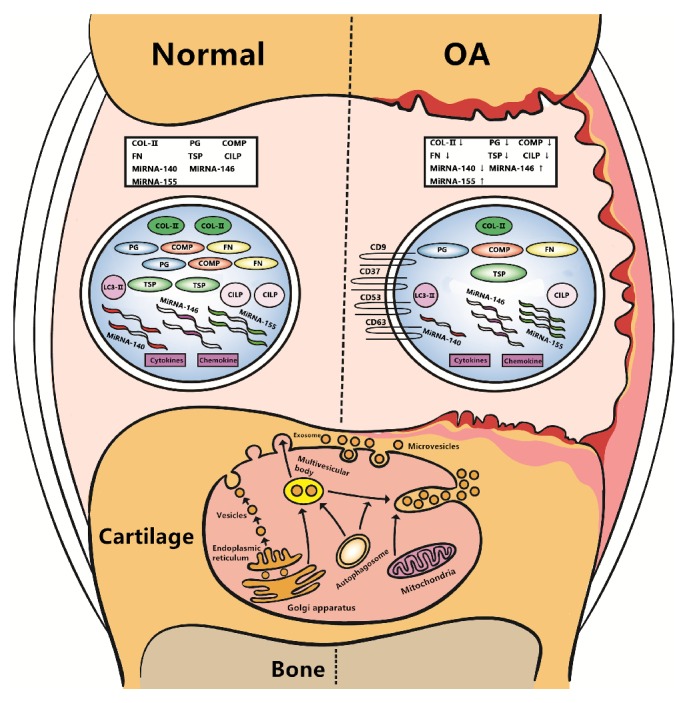
Some differences in the proteome and miRNA content in ACVs between OA and normal cartilage. These important findings are likely to become the basis for future accurate diagnoses and raise diagnosis precision to the molecular level in OA. The content of collagen type II, proteoglycan (PG), cartilage oligomeric matrix protein (COMP), fibronectin protein (FN), thrombospondin (TSP), and cartilage intermediate layer protein (CILP) in ACVs from a normal person is much higher than that from OA patients. The miRNA-140 content in OA patients is much lower than that in a normal person, while the miRNA-146 and miRNA-155 contents were found to be higher in OA patients than in normal persons.

There are some differences in the proteome content of ACV between OA and normal cartilage, and these differences may be largely ascribed to the component changes of the ECM in OA cartilage (Figure 1). Some of the ECM protein content of ACV in normal persons is much higher than that in OA patients, including collagen type II, some proteoglycans (PG), including biglycan, proline/arginine-rich, and leucine-rich repeat proteins, aggrecan and perlecan, cartilage oligomeric matrix protein (COMP), fibronectin protein (FN) and thrombospondin (TSP), and cartilage intermediate layer protein 1 and 2 (CILP 1,2) [23]. From this phenomenon, we can speculate that ECM destruction may cause the decrease in these important proteins in OA patients. In the future, we can detect the content of these important proteins to observe more sensitively the early occurrence of OA from a molecular biology perspective. Moreover, early intervention measures are warranted that delay or prevent the further development of early-stage OA.

As one type of vesicle carrier, EV can effectively protect the RNA sequence from rapid degradation by RNA hydrolase, allowing them to be safely transported to the extracellular space (Figure 1). EVs as carriers of these key miRNAs also play a role in the information exchange between cells. Expression of miRNA-140 [54], a very small RNA with high expression in chondrocytes, in OA patients was found to be much lower than that in normal persons, indicating that it may play an important role in regulating cartilage homeostasis. In addition, the expression of disintegrin, metalloproteinase, thrombospondin motifs 5, and aggrecan core protein was regulated by miRNA-140, also indirectly illustrating that miRNA-140 may play a regulatory role in maintaining the ECM balance between synthesis and degradation. miRNA-146 [55] is also highly expressed in the early stage of OA, and its expression can be affected by IL-1*β*. It was shown that miRNA-146 may also play an important role in OA. miRNA-155, an autophagy-related miRNA, was found to have a high expression in OA. It can suppress chondrocyte autophagy in OA by modulating the expression of autophagy-related proteins [56]. These important findings are likely to become the basis for future accurate diagnoses and raise diagnosis precision to the molecular level in OA. Moreover, we can determine the extent of disease progression more accurately that heretofore, and make the most appropriate treatment plan in relation to these small molecule changes.

## 7. Treatment of OA Using EV

Some ACV-related ECM proteins that mediate collagen production show that ACVs also have a matrix repair function [57]. ACVs have intercellular signal channel recognition mechanisms that can respond to exogenous signals. Some growth factors, such as BMP, are helpful in regulating chondrocyte phenotypes [54]. Chondrogenesis factors, including BMP-3, angiogenesis-related proteins, and melanoma suppressor proteins, are also present in ACV. MV isolated from growth plate cartilage shows high BMP expression. In rat ectopic chondrogenesis experiments, it was found that chondrocytes can secrete BMP-2, and then there are some vessel-like structures formed [58]. The MVs that carry BMP are released to the matrix in the hypertrophic region of the growth plate. After the beginning of mineralization, the MV membrane ruptures, and BMP is released into the growth plate matrix. BMP sourced from MV can induce the differentiation of adjacent chondrocytes, recruiting more osteoblasts and osteocytes gathering in the metaphysis. It can also promote the resorption of cartilage matrix and make the growth plate cartilage convert to bone trabecula in the metaphysis [21]. Multinuclear osteoblasts can absorb mineralized cartilage matrix, thus leading to the creation of a support structure for sedimentary osteoblasts and the early formation of cancellous bone.

The paracrine effect of mesenchymal stem cells (MSCs) was first proposed in 1996 [59], and MSC synthesis and secretion of a wide range of growth factors, chemokines, and cytokines can greatly influence cells that are adjacent to them. At the same time, MSCs can secrete various types of EV, such as microvesicles and EXO. Stem-cell derived EV loaded with various endogenous RNAs and proteins can cause rearrangement of the gene sequence, inducing cell proliferation, guiding cell cycle reboot, and starting tissue regeneration [60]. In addition to endogenous substances, some exogenous biomaterials can be loaded into EXO: in vivo drug loading: the genes that encode for the RNA/protein of interest are introduced into exosome-secreting cells by standard gene transfection methods; in vitro drug loading: the drugs of interest are loaded into purified exosomes using standard membrane breaching techniques such as electroporation and lipofection [61]. Thus we can believe that it will provide the possibility of accurate treatment by EXO-loaded drugs in the future.

Among the various types of EV, EXO has many advantages compared with other drug carriers: first, EXOs have a strong ability to resist biological degradation in vivo, and they are also widely distributed among the various types of body fluids, such as blood, saliva, urine, and synovia, even including milk [62]; second, EXO has long cycle times—they can be precisely recognized by target cells, transport the carried substances to target tissue smoothly [63], and then plant in the target tissue; third, EXO carries many macromolecules such as miRNA, DNA, and proteins, leading to a therapeutic effect on cells [64, 65].

**Figure 2:**
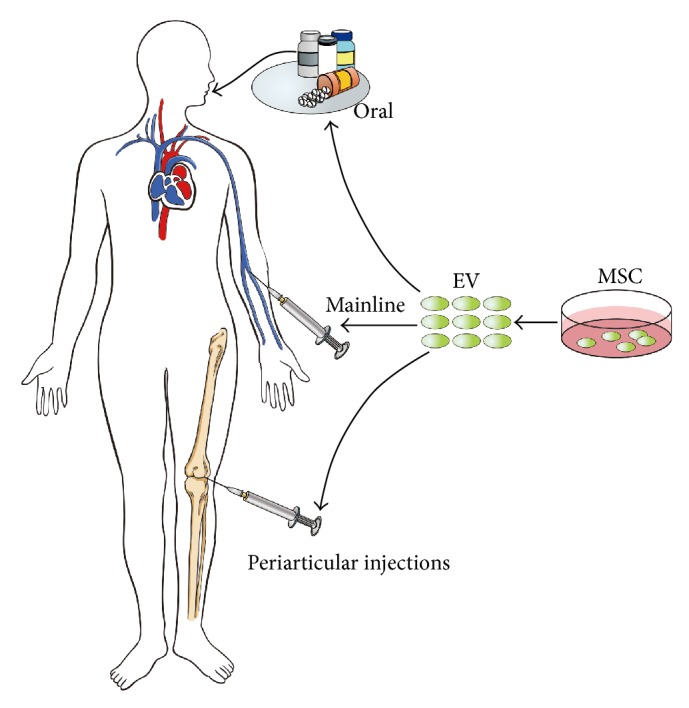
Potential therapeutic strategies using EV to treat OA. EV could be abundantly obtained from MSC in vitro; then it will be processed by different approaches in order to achieve various methods of administration, such as oral medication, the periarticular injections, or system injections.

There are multiple potential therapeutic strategies using EV to treat OA (Figure 2). In addition to the periarticular injections of EV, system injections also have some therapeutic effects [61]. EVs play a role in regulating cell recruitment and cell proliferation and differentiation; therefore, EVs also have the potential to induce tissue regeneration. The latest studies have shown that, in animal experiments, milk-derived EXO can delay RA development by oral administration [63], possibly providing a new perspective, to some extent, for resolving the problem of the source of EV.

## 8. Conclusion

With the advent of an aging society, the incidence of OA also shows a rising trend. The long-term pain associated with OA patients is no less than that associated with cancer. There are many theories that can explain the pathogenesis of OA; however, there has been no systematic and complete conclusion to date. Hence, elucidating the pathogenesis of OA is an indispensable step for the early diagnosis and treatment of OA. In recent years, more and more attention has been paid to EV, including MV and EXO. It is unknown which organelles will produce ACV, and not many factors will affect the synthesis and release of ACV. ACVs have some special characteristics that are thought to make them and their content beneficial to an OA early diagnosis. However, there is no doubt that ACV plays a critical role in the mineralization of bone and cartilage. Although the role of chondrocyte dysfunctional autophagy in OA pathogenesis has not been completely explored, further understanding of the impact of autophagy in OA may offer a new train of thought in generating OA treatment strategies. Before EXO can be used as a drug carrier in clinical settings, some problems need to be resolved, and further research is warranted in terms of their biocompatibility, use as an economically viable source, methods to obtain EXO, therapeutic effects, and tolerance in vivo. In the future, EXO combined with autophagy-related drugs might open up another innovative research field relating to OA therapeutic strategies.
